# Heat‐labile *Escherichia coli* toxin enhances the induction of allergen‐specific IgG antibodies in epicutaneous patch vaccination

**DOI:** 10.1111/all.13036

**Published:** 2016-09-30

**Authors:** C. R. Cabauatan, R. Campana, K. Niespodziana, C. Reinisch, U. Lundberg, A. Meinke, R. Henning, A. Neubauer, R. Valenta

**Affiliations:** ^1^ Division of ImmunopathologyDepartment of Pathophysiology and Allergy ResearchCenter for Pathophysiology, Infectiology and ImmunologyMedical University of ViennaViennaAustria; ^2^ Valneva Austria GmbHCampus Vienna BiocenterViennaAustria; ^3^ Biomay AGViennaAustria

**Keywords:** allergen‐specific immunotherapy, epicutaneous allergen‐specific immunotherapy, heat‐labile *Escherichia coli* toxin, patch delivery system, rBet v 1

## Abstract

Epicutaneous allergen‐specific immunotherapy (EPIT) is proposed as an alternative route for allergen‐specific immunotherapy (AIT). The induction of allergen‐specific blocking IgG antibodies represents an important mechanism underlying AIT, but has not been investigated for EPIT. Here, we compared the induction of allergen‐specific blocking IgG in outbred guinea pigs which had been immunized with recombinant birch pollen allergen Bet v 1 using patch delivery system (PDS) with or without heat‐labile toxin (LT) from *Escherichia coli* or subcutaneously with aluminum hydroxide (Alum)‐adsorbed rBet v 1. Only subcutaneous immunization with Alum‐adsorbed rBet v 1 and epicutaneous administration of rBet v 1 with PDS in combination with LT from *E. coli* induced allergen‐specific IgG antibodies blocking allergic patients' IgE, but not immunization with rBet v 1 via PDS alone. Our results suggest that patch vaccination with rBet v 1 in combination with LT may be a promising strategy for allergen‐specific immunotherapy against birch pollen allergy.

Epicutaneous AIT (EPIT) has been suggested as an alternative route of administration for allergen‐specific immunotherapy (AIT), because it is a needle‐free treatment, offers the possibility of self‐administration, and may allow targeting professional antigen‐presenting cells (i.e., dendritic cells, Langerhans cells) residing in the skin 1, 2. EPIT has been shown to be clinically effective in allergic patients 3, 4, but its immunological mechanisms have not been studied. Several studies performed in animals have shown that EPIT has immune modulatory effects on allergen‐specific T‐cell responses 5, 6. In these animal studies, it has been mainly investigated what effects EPIT has on established allergic immune responses in animals which had been sensitized before treatment, but not the effects of EPIT on the immune system as such 5, 6. It is unknown whether EPIT induces allergen‐specific IgG antibodies and whether such allergen‐specific IgG antibodies are able to block allergic patients' IgE binding to the allergen. The latter is of interest, because the induction of allergen‐specific blocking IgG is one major mechanism in successful AIT 7.

In this study, we have tested a patch delivery system (PDS) as a technique for transcutaneous immunization (TCI) which has been developed and clinically tested for vaccination of travelers' diarrhea which is caused by enterotoxigenic *Escherichia coli* (ETEC) producing heat‐labile enterotoxin (LT) 8. Here, we used recombinant major birch pollen allergen (rBet v 1) as a model allergen to compare epicutaneous administration of the allergen with and without LT as adjuvant via PDS with classical immunization based on subcutaneous injection of Alum‐adsorbed rBet v 1 regarding the induction of allergen‐specific blocking IgG in outbred guinea pigs.

## Methods

### Animals and study design

**Figure 1:**
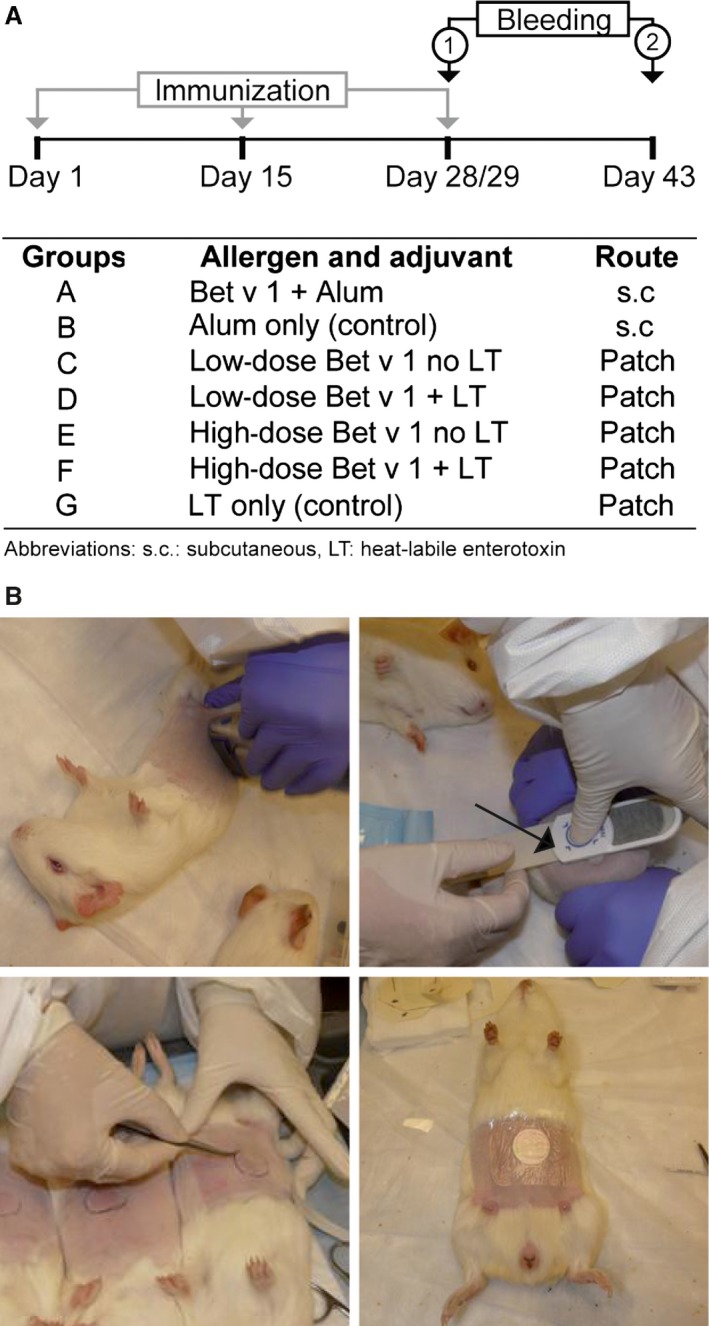
Study design. Time course of immunizations and bleedings for the groups of guinea pigs (groups A–G) receiving different treatments, allergens, and/or adjuvants subcutaneously or via patch‐administered vaccine (A). Illustration of the patch delivery system application to guinea pigs. Shaving, pretreatment using skin preparation system (SPS) device, application of patch, and securing of patch (from upper left to upper right, lower left, and lower right) (B).

All animal experiments were performed in accordance with Austrian law (BGB1 No. 114/2012) and were approved by ‘Magistratsabteilung 58’ of the city of Vienna, Austria. Eight‐week‐old outbred, female Dunkin Hartley guinea pigs (ten animals/group) with a body weight (BW) range between 500 and 550 g were studied. Group A was immunized s.c. with 10 μg rBet v 1 (Biomay AG, Vienna, Austria) adsorbed to 200 μL of 100 μg/mL aluminum hydroxide (i.e., 20 μg Alum; Brenntag, Mülheim an der Ruhr, Germany), whereas group B received 200 μL Alum alone (Fig. 1A). Patch‐immunized groups (groups C–G) were administered 30 μg rBet v 1 (low dose) without LT (group C) or with 5 μg LT (group D) or 100 μg rBet v 1 (high dose) without LT (group E) or with 5 μg LT (group F). Group G was administered only 5 μg LT without allergen. All immunizations were done on days 1, 15, and 28 (Fig. 1A).

The patch was formulated with dried bulk stabilizing solution (12% w/v sucrose, 8% w/v maltitol, 0.1% w/v poloxamer 188, 10 mM sodium phosphate, pH 7.2) containing the antigen and adjuvant (allergen, LT). The stability of the patch formulation was monitored by RP‐HPLC analysis and CD spectroscopy for 6 months.

For the administration of the PDS, and for blood collection, guinea pigs were intraperitoneally anesthetized with ketamine (60 mg/kg BW) and xylazine (2 mg/kg BW). Fur on the abdomen between the sternum and hind legs was shaved (Fig 1B, upper left) followed by pretreatment with sandpaper (180 grit; 3M, St. Paul, MN, USA) which is integrated in the Skin Preparation System (SPS) device (Valneva Austria GmbH, Vienna, Austria; Fig 1B, upper right, SPS is marked with an arrow). Dry circular rayon/cellulose matrix patch (19.05 mm in diameter and with 2.85 cm^2^ surface area, Valneva Austria GmbH) was then placed on the abraded skin (Fig 1B, lower left) and secured with a transparent film dressing (3M) for at least 12 hours (Fig. 1B, lower right). The trans‐epidermal water loss as measured with the DermaLab modular system with trans‐epidermal water loss probes (Cortex Technology, Hadsund, Denmark) was 6–9 g/m^2^/h before pretreatment and 60–80 g/m^2^/h after treatment with the SPS device.

Blood samples were drawn via ear punch for the interim bleeding (day 28/29), while terminal bleeding was taken from the retrobulbar plexus at day 43. From groups A, C, and E, terminal blood samples were collected from 9 of the 10 animals and available for analysis.

### Measurement of LT‐specific and allergen‐specific antibody responses and of the ability of guinea pig antibodies to inhibit allergic patients' IgE binding to rBet v 1 by ELISA

The measurement of specific antibody responses and of the ability of guinea pig antibodies to inhibit allergic patient's allergen‐specific IgE binding was performed by ELISA and inhibition ELISA, respectively (see Data S1).

## Results and discussion

**Figure 2:**
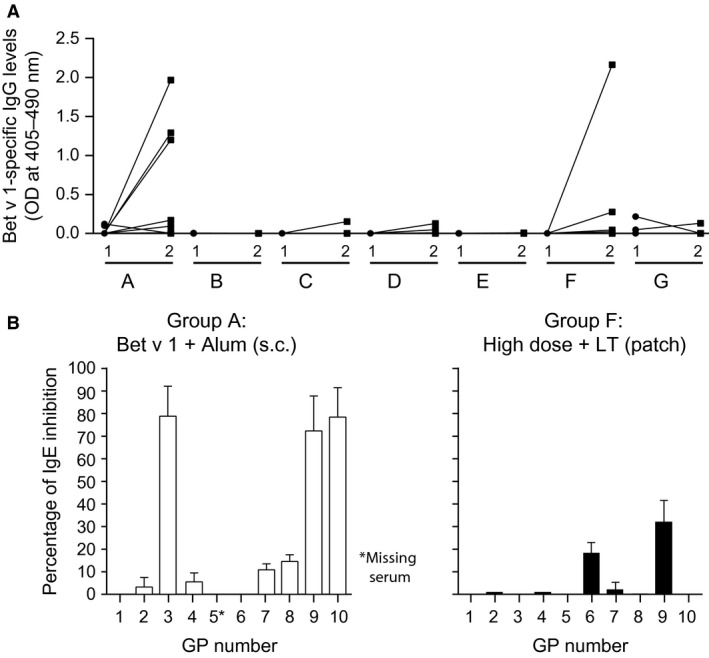
Induction of blocking IgG antibodies in guinea pigs. Shown are rBet v 1‐specific IgG levels on day 28/29 (1) and day 43 (2) as optical densities (*y*‐axis) for each of the groups (*x*‐axis: groups A–G) (A). Mean percentage inhibition ±SDs of allergic patients' (Patients 1–5) IgE binding to rBet v 1 (*y*‐axes) by antibodies from guinea pigs (*x*‐axes: 1–10) immunized with Alum‐adsorbed rBet v 1 (left panel: white bars) or with high‐dose rBet v 1 + LT (right panel: black bars (B)).

In this study, we used rBet v 1 to compare a PDS which has been developed for epicutaneous vaccination against travelers' diarrhea with subcutaneous immunization based on Alum‐adsorbed allergen regarding the induction of allergen‐specific IgG blocking antibodies in a guinea pig model. The allergen doses selected for epicutaneous and subcutaneous vaccination were similar to the ones used in clinical trials performed in allergic subjects 3, 9 and in an animal EPIT study 6. Outbred guinea pigs were used because guinea pig skin has similar characteristics regarding thickness and permeability as human skin 10 and because a PDS of the same size can be used also for humans (Fig. 1B). We found that only subcutaneous immunization with Alum‐adsorbed rBet v 1 and patch vaccination with a high dose (i.e., 100μg/patch) of rBet v 1 together with LT induced relevant allergen‐specific IgG responses, whereas the low‐dose patch administration of rBet v 1 (i.e., 30 μg/patch) and the high dose without LT did not induce relevant rBet v 1‐specific IgG levels (Fig. 2). We noted that not all animals of the group which had been immunized with Alum‐adsorbed rBet v 1 and of the high‐dose LT group developed high levels of rBet v 1‐specific IgG. This may be due to the fact that IgG responses were assessed already on day 43 after only three vaccinations and/or due to the fact that outbred guinea pigs are poor responders for the rBet v 1 allergen. However, we found that sera from each of the animals with robust rBet v 1‐specific IgG responses inhibited the binding of allergic patients' IgE to the rBet v 1 allergen (Fig. 2B). This result is important because patch vaccination has not yet been shown to induce allergen‐specific blocking IgG. Furthermore, the epitope specificity of such IgG has not been characterized and compared with that of allergen‐specific IgG induced by subcutaneous immunotherapy 7. The analysis of the antibody response showed that those guinea pigs which had received rBet v 1 together with LT on the patch also developed LT‐specific IgG responses (Fig. S1). We think that the addition of LT is important for the induction of allergen‐specific IgG, because only animals from the high‐dose LT group but not from the group receiving only the high dose without LT developed robust levels of allergen‐specific blocking IgG. This is interesting because in a clinical study performed in allergic patients, we found that epicutaneous application of a high dose of rBet v 1 (i.e., 160 μg/patch) only induced allergen‐specific T‐cell responses but no relevant allergen‐specific IgG (NCT02098551; Campana & Valenta, unpublished data). There are several possibilities how LT may enhance allergen‐specific IgG production as an adjuvant: It may attract and directly stimulate B cells 11. Alternatively, it may retain intact antigen on the surface of dendritic cells, because it was found that LT‐treated DCs showed an impaired antigen presentation capacity due to a slower rate of endocytosis 12. Prolonged retention of intact antigen on the surface of DCs may then allow B cells to recognize the DC‐bound antigen by their immunoglobulin receptors. Both possibilities are supported by our finding that IgG induced by patch vaccination only recognized the intact, folded and complete rBet v 1 allergen, but not unfolded recombinant rBet v 1 fragments (Fig. S2) 13. By contrast, IgG antibodies induced by immunization with Alum‐adsorbed rBet v 1 also recognized unfolded rBet v 1 fragments (Fig. S2). The Alum‐rBet v 1‐induced antibodies also showed a broader cross‐reactivity to rBet v 1‐related pollen and food allergens (Fig. S2).

Our study has some limitations such as the short period of immunization, that it was performed only once and that the IgG responses in the animals of the rBet v 1‐Alum and high‐dose rBet v 1‐LT groups were not uniform. However, this was compensated by the representative numbers of animals per group (i.e., 10/group) and the inclusion of different doses and control groups (i.e., LT and Alum alone). In fact, our study shows in outbred animals that LT enhances the production of allergen‐specific IgG during EPIT and thus seems to be a useful adjuvant for EPIT. In fact, it was found that even much higher doses of LT caused only mild local reactions in clinical studies and therefore LT is also a safe adjuvant 8. Furthermore, the PDS developed for vaccination of travelers' diarrhea is a highly standardized device which may be evaluated for EPIT for all kinds of allergen sources and in particular for defined recombinant allergens in clinical studies.

## Author contributions

A. Meinke and R. Valenta contributed to the overall design and supervision of the study. C. Reinisch formulated and prepared the patches, while U. Lundberg handled animal experiments. R. Henning and A. Neubauer provided the recombinant allergens. C. R. Cabauatan did serological analyses with well‐characterized sera from birch pollen allergic patients provided by R. Campana and also with control guinea pig sera and proteins given by K. Niepodziana. C. Cabauatan, and R. Valenta analyzed data, interpreted data, and prepared the manuscript which was reviewed by each of the other authors.

## Funding

This study was supported by grant F4605 of the Austrian Science Fund and a research grant from Biomay AG, Vienna, Austria.

## Conflicts of interest

R. Valenta has received research grants from Biomay AG, Vienna, Austria, and serves as a consultant for this company. R. Heining and A. Neubauer are employees of Biomay AG. C. Reinisch, U. Lundberg, and A. Meinke were former employees of Intercell, Austria, and are currently employees of Valneva Austria GmbH, Vienna, Austria.

## Supporting information


**Figure S1.** LT‐specific IgG antibodies. Box plots (means: horizontal bars; whiskers = minimum and maximum; boxes = 25th to 75th percentiles) showing the half‐max titers (log 10, y‐axis) of LT‐specific IgG for guinea pigs from the different groups (x‐axis).

**Figure S2.** IgG reactivity (y‐axes: OD values corresponding to IgG levels) of guinea pigs immunized subcutaneously with Alum‐adsorbed rBet v 1 (left panel: 1‐4, 6‐10) or by patch‐administered high dose rBet v 1 + LT (right panel: 1‐10) to rBet v 1 fragments F1 and F2 (upper panel) and to cross‐reactive allergens (Aln g 1, Cor a 1, Mal d 1) (lower panel).

**Data S1.** Methods
