# Supplementary material for: Heat‐labile Escherichia coli toxin enhances the induction of allergen‐specific IgG antibodies in epicutaneous patch vaccination
Source: Allergy. 2016 Sep 30;72(1):164–8. doi: 10.1111/all.13036 (PMC5215485; doi:10.1111/all.13036)
Supplement: Supplementary file 3 — Data S1. Methods [file ALL-72-164-s003.doc]

**Supplemental Materials**

**Measurement of LT-specific and allergen-specific antibody responses and of the ability of guinea pig antibodies to inhibit allergic patients’ IgE binding to rBet v 1 by ELISA**

Ninety-six well ELISA plates (Maxisorp, Nunc, Denmark) were coated with antigens (50 ng LT/well, Sigma-Aldrich, St. Louis, MO; 500 ng/well rBet v 1 fragment F1 aa 1-74, F2 aa 75-160 (E1); 500 ng/well rBet v 1, rAln g 1, rCor a 1 or Mal d 1, Biomay AG) dissolved in phosphate buffered saline (PBS) at 4°C for 16 to 20 h. Plates were washed with PBS-T (PBS + 0.05% Tween-20) and blocked with 1% BSA/PBS-T (PBS-T + 0.1% [w/v] bovine serum albumin). For the measurement of LT-specific antibodies, five-fold dilutions of GP sera were added and incubated for 1 h at room temperature (RT). After washing, 1:2,000 diluted (in 0.5% BSA/PBS-T) HRP-conjugated (Horseradish peroxidase-conjugated) donkey anti-GP IgG (Fitzgerald Industries International, USA) was added. For the measurement of allergen-specific IgG antibodies, guinea pig sera were diluted 1:500, added to the plates for 2 hours at RT, washed and bound IgG was detected with HRP-conjugated goat anti-GP IgG (1:10,000 diluted, Jackson ImmunoResearch Laboratories, USA). HRP-labelled antibodies were visualized with ABTS (2,2'-azino-bis(3-ethylbenzothiazoline-6-sulfonic acid)) (Sigma-Aldrich) as substrate and absorbance was measured at 405 nm (Synergy 2, BioTek, USA). The half-maximum titer of LT-specific antibodies was computed with the software Gene5 (BioTek). Mean levels of allergen-specific IgG correspond to mean OD levels from triplicate determinations.

For the IgE-inhibition ELISA, rBet v 1-coated ELISA plates (20 ng/well) were incubated with GP sera (1:10 in PBS) or PBS alone and, after washing, with sera from patients with birch pollen allergy (n=5, 1:5 diluted). Bound IgE is then detected with a HRP-conjugated goat anti-human IgE (1:2,500 in PBS-T) (KPL, Gaithersburg, MD). Optical density (405-490 nm) corresponding to bound antibodies were recorded in an ELISA reader (Molecular Devices, Sunnyvale, CA). The percentage inhibition of IgE binding was calculated as follows: Percent inhibition = 100 – (OD^a^ x 100/ OD^b^). OD^a^ and OD^b^ represent the extinctions after pre-incubation with GP serum and buffer alone, respectively. Shown are mean percentages of inhibition for the five patients +/- SDs.

REFERENCES

E1. Vrtala S, Hirtenlehner K, Vangelista L, Pastore A, Eichler HG, Sperr WR et al. Conversion of the major birch pollen allergen, Bet v 1, into two nonanaphylactic T cell epitope-containing fragments: candidates for a novel form of specific immunotherapy. *J Clin Invest* 1997;**99**:1673-1681.
